# Parameter Optimization for Selected Correlation Analysis of Intracranial Pathophysiology

**DOI:** 10.1155/2015/652030

**Published:** 2015-11-29

**Authors:** Rupert Faltermeier, Martin A. Proescholdt, Sylvia Bele, Alexander Brawanski

**Affiliations:** Department of Neurosurgery, University Hospital Regensburg, 93042 Regensburg, Germany

## Abstract

Recently we proposed a mathematical tool set, called selected correlation analysis, that reliably detects positive and negative correlations between arterial blood pressure (ABP) and intracranial pressure (ICP). Such correlations are associated with severe impairment of the cerebral autoregulation and intracranial compliance, as predicted by a mathematical model. The time resolved selected correlation analysis is based on a windowing technique combined with Fourier-based coherence calculations and therefore depends on several parameters. For real time application of this method at an ICU it is inevitable to adjust this mathematical tool for high sensitivity and distinct reliability. In this study, we will introduce a method to optimize the parameters of the selected correlation analysis by correlating an index, called selected correlation positive (SCP), with the outcome of the patients represented by the Glasgow Outcome Scale (GOS). For that purpose, the data of twenty-five patients were used to calculate the SCP value for each patient and multitude of feasible parameter sets of the selected correlation analysis. It could be shown that an optimized set of parameters is able to improve the sensitivity of the method by a factor greater than four in comparison to our first analyses.

## 1. Introduction

The course of severe neurological events like subarachnoid hemorrhage (SAH) and traumatic brain injury is influenced by two main pathophysiological principles: (A) the primary injury sustained at the time of impact which is mostly irreversible and therefore not primary object to treatment [1], (B) the secondary injury consisting of cytotoxic and vasogenic edema with increased intracranial pressure (ICP), reduced cerebral blood flow with consecutive brain ischemia, and insufficient oxygenation leading to programmed cell death of neurons that can be detected from hours to days following injury and may contribute to neurological dysfunction [2–4]. The primary goal of neurointensive care treatment is therefore to avoid secondary brain injury by providing an optimal physiological and biochemical environment [5]. Since the biological changes leading to secondary injury are highly individual [6], a recent consensus has defined the necessity for patient specific treatment protocols in contrast to a rigid all size fits all approach [7]. In this circumstance, the cerebral pressure autoregulation maintaining a continuous cerebral blood flow despite variations of systemic arterial pressure is of paramount importance [8, 9]. Under physiological conditions, an increase of ABP will not induce higher ICP levels. However, if the autoregulation disturbed, a positive correlation between ABP and ICP will occur [10]. Therefore, if the autoregulation is intact, enhancing the systemic blood pressure leads to improved cerebral perfusion pressure (CPP) and appropriate cerebral blood supply. Conversely, in a patient with impaired autoregulation, augmentation of CPP may cause brain swelling and worse outcome [11]. Recent studies indicated that a deviation from the putatively optimal CPP based on the function of cerebral autoregulation will lead to significantly worse outcome of the patients [12]. Therefore an individualized treatment strategy accounting for the autoregulation status of the patient is necessary [13]. This however requires an array of different monitoring techniques for the assessment of intracranial pressure (ICP), oxygenation status, and metabolism [14, 15] leading to an immense volume of multimodal datasets frequently overwhelming the treating physician [16]. To address this problem, we have recently developed a mathematical tool set termed selected correlation analysis that unmasks deterioration of the cerebral autoregulation [17] and indicates reduced intracranial compliance [18]. However, this approach needs to be validated in a prospective study allowing the adjustment of treatment ultimately leading to improved outcome of the affected patients [14]. The goal of our study was to optimize the parameter of selected correlation analysis in order to provide the most sensitive and specific tool set for a randomized clinical trial assessing the benefit of the proposed method.

## 2. Methods

### 2.1. Patient Population

The study was conducted in accordance with the ethical guidelines of the University of Regensburg Institutional Review Board. Informed consent was obtained from the patient's relatives; all study results were stored and analyzed in an anonymized fashion. We prospectively investigated a cohort of 25 adult patients (13 females, 12 males) who were treated at the neurosurgical intensive care unit for traumatic brain injury (TBI) or subarachnoid hemorrhage in 9 and 16 cases, respectively. We have exclusively included patients with critical neurological diseases in our study since only in this patient subgroup multimodal brain monitoring is clinically indicated. The mean age was 43.4 years (range: 18.4–72.4); the median Glasgow Coma Scale (GCS) at the time of admission was 6 (range: 3–15). Follow-up was completed up to January 2015 by reviewing outpatient records and contacting the patient's family member or the patient's primary physician. The mean follow-up time was 39.8 months; no patient was lost to follow-up. The neurological outcome was measured by the Glasgow Outcome Scale at last follow-up; the median score last follow-up was 3 (range: 1–5). All patients were sedated and mechanically ventilated during the observation period and received an intra-arterial catheter for the continuous measurement of arterial blood pressure as part of the standard treatment procedure in our institution. ICP monitoring was performed continuously using either an external ventricular drain equipped with an electronic pressure device (EVD) or a parenchymal ICP probe (both from Raumedic, Helmbrechts, Germany). The ABP and ICP data were acquired continuously using a data logger (Daq USB 6210, National Instruments, Munich, Germany) with a sample frequency of 1000 Hz. For the correlation analysis, the data were resampled to 0.2 Hz (one data point every five seconds) to reduce noise effects and to smooth out fast oscillations or spikes. Additionally, the above-mentioned resampling rate ensures that the low homeostatic variations of the data are contained within the window sizes we will discuss.

### 2.2. Correlation Index Calculations

In the following we will roughly sketch the mathematical framework used by selected correlation analysis. For a more detailed description of the different applied characteristics, especially the calculation of the error rates, please see [17].

To identify the above-mentioned positive correlation between ABP and ICP in monitoring data from the ICU, we use a windowing approach combined with the multitaper method (mtm [19]) to determine the coherence between segments of two time series that were synchronously recorded with a sampling rate of 0.2 Hz. From the isochronous time series *A*, *I* we select windows *w*_*k*_^*A*^, *w*_*l*_^*I*^ of fixed size *s* with *s* = 2^*z*^, *z* ∈ *ℕ*, and potentially different starting points *k* and *l* and then calculate the mtm-spectra mtms(*f*_*i*_) and the mtm coherence between the windows (mtmc(*f*_*i*_)):

(1) wkA ≔xk,…,xk+s−1,wlI≔yl,…,xl+s−1,SlIfi ≔ mtmsfi  of  wlI,Ck,lA,Ifi ≔ mtmcfi  of  wkA,wlI.

As the mtm provides a built-in significance test, each single frequency *f*_*i*_ is tested for significance. Building on this, we define the pointwise selected correlation (PSC) assuming a fixed significance level *C* for the built-in significance test:

(2) PSCk,lA,I ≔psc1A,Ik,l,…,pscs/2A,Ik,l  with:psciA,Ik,l ≔1,if  SkAfi,SlIfi,Ck,lA,Ifisig.,0,otherwise.

The requirement of being significant for a frequency *f*_*i*_ in both spectra guarantees that only frequencies are considered that essentially contribute to the original signals, whereas, in case of the coherence the requirement assures that specific *f*_*i*_ exhibits a correlation between the input signals. Repeating the PSC calculations for *N* pairs of isochronous windows leads to the mean pointwise selected correlation (MPSC):

(3) $$\begin{array}{c} {MPSC}^{A,I}=\overset{j=N}{\underset{j=1}{\sum }}{PSC}^{A,I}\left(\;j,j\;\right). \end{array}$$

The elements of the MPSC list represent the percentage of a significant occurrence in both spectra and the coherence calculation for each single frequency *f*_*i*_. With MPSC we are able to determine frequency intervals that contain relevant correlations within a whole dataset. After having identified such a frequency interval *U* = (*f*_*m*_,…, *f*_*n*_) by examining several different datasets, we want to determine periods in the dataset where strong correlation with respect to *U* occurs. Therefore we first estimate the degree of correlation of a distinct pair of windows with respect to *U* by calculating the sum of all elements psc_*k*_ of PSC belonging to the frequency band *U*. This sum divided by the length of *U* is called selected correlation (sc):

(4) scm,nA,Ik,l≔1n−m+1∑j=mj=npscjA,Ik,l0≤m<n≤s2.

A pair of windows is called selected correlated if sc > *l*sc for a predefined threshold *l*sc. The sc value therefore serves as a measure for the degree of correlation of a pair of data windows with respect to a specific frequency range *U*. To obtain time resolved information about the selected correlation we determine the index sc_*m*,*n*_^*A*,*I*^(*l*, *l*) for isochronous windows while shifting the starting point *l* along the time axis. Additionally we use a statistical test, calculating error rates of false positives, to determine the significance of the threshold *l*sc.

### 2.3. Statistical Test

The statistical test for significance of *l*sc, a kind of perturbation test, is based on the model prediction of isochronous correlations between ABP and ICP. Two segments should not be correlated if their starting points are quite apart from each other. Assuming that a sc value is meaningful if higher predefined threshold *l*sc, we can count how often these separated windows produce sc values higher than *l*sc. The amount of wrong hits is interpreted as the error rate of sc with respect to *l*sc and therefore determines the significance of sc with respect to *l*sc. To identify a sufficient offset for the input window we use the so-called mean windowed autocorrelation (mwa):

(5) $$\begin{array}{c} {mwa}^{A}\left(\;o\;\right)≔\left(\;\frac{1}{E}\;\right)\overset{e=E}{\underset{e=1}{\sum }}{sc}_{m,n}^{A,A}\left(\;e,e+o\;\right). \end{array}$$

If the time shift *o* is large enough to exclude autocorrelation artifacts, the subsequent mwa values should be small and stable. With this offset we can calculate the error index, ei_*m*,*n*_^*A*,*I*^(*a*, *b*) indicating whether the selected correlation sc_*m*,*n*_^*A*,*I*^(*a*, *b*) is higher than a predefined limit *l*sc, and the error rate *a*sc, that is, the rate of obviously wrong hits with respect to *l*sc:

(6) eim,n,lscA,Ia,b ≔1,if  scm,nA,Ia,b>lsc,0,otherwise,ascm,nA,Ilsc ≔1K∑i=1i=Keim,n,lscA,Iai,bi.

Accordingly a pair of data segments is called significantly correlated if the sc value of this pair is higher equal to the predefined limit *l*sc. The significance of this correlation is specified by the appropriate error rate.

### 2.4. Hilbert Phase Differences

Having identified a pair of windows exhibiting sufficient high correlation index sc, we have to determine the phasing between the two data windows. This is done by calculating the mean Hilbert phase difference (mhpd) of the corresponding data segments, leading to values of mhpd between 0 and 180 deg [17]. The above-described error rate calculations for the sc value can easily be adapted to calculate the error rates of mhpd by substituting the *l*sc criterion with appropriate criteria called *l*mhpd_pos_. If mhpd < *l*mhpd_pos_ the correlation between the data will be called positive.

### 2.5. Parameter Optimization

With the above-described tools we are now able to calculate the percentage of pairs of windows that are significantly positive correlated for each individual patient. This percentage is called selected correlation positive (SCP). As SCP describes the percentage of measurement time in which the cerebral regulatory systems, autoregulation, and compliance are distinctively disturbed, this index is a reliable predictive value for the patients outcome [17]. But the magnitude of an individual SCP depends on several parameters needed by the above-mentioned mathematical tools. In detail this parameter is the significance of the mtm built-in statistical test, the window size of the data pairs, the frequency interval *U* used for the sc calculations and the limits, *l*sc, and *l*mhpd_pos_ for the selected correlation and the mean Hilbert phase of the data. To find the best set of parameters we first vary all parameter belonging to sc in some natural limits (see Table 1) and calculate for each resulting parameter set the SCP for each patient assuming a *l*mhpd_pos_ of 50 deg, an appropriate offset for the error rate calculations as used in our previous study [17]. Then we determine the predictive capability of a specific parameter set with respect to our patient cohort by calculating the *p* value of the Pearson correlation between the patients SCP and GOS. Additionally, we calculate a parameter called yield, which is the SCP value of the complete dataset. In other words, yield describes the sum of all SCP values derived from the entire patient population weighted by the patients individual observation time and therefore serves as a measure of the sensitivity of the method. Having found an optimal set of parameters for the sc calculations we subsequently vary *l*mhpd_pos_ for this fixed sc parameter set and test the impact on SCP and yield exactly as described above.

## 3. Results

**Figure 1:**
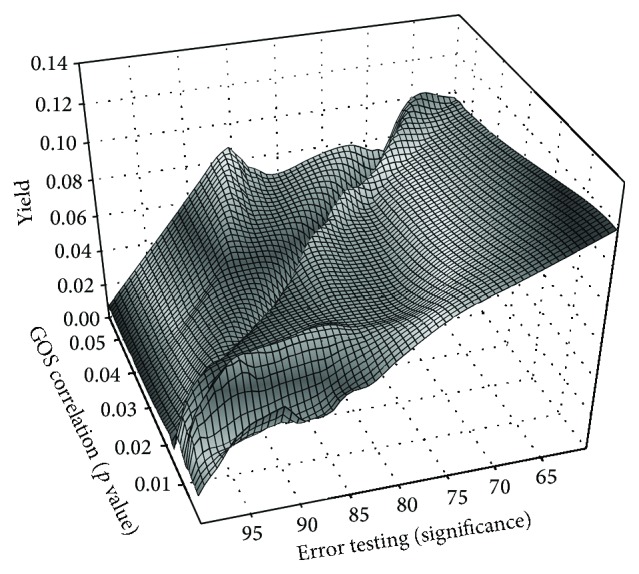
3D mesh graph illustrates the variability of SCP detection in correlation with the parameter settings: *x*-axis displays the *p* value of a Pearson correlation between the relative time of SCP per observation time and clinical outcome measured by the Glasgow Outcome Scale; *y*-axis shows the significance of error testing; *z*-axis shows the yield (frequency of SCP detection).

**Figure 2:**
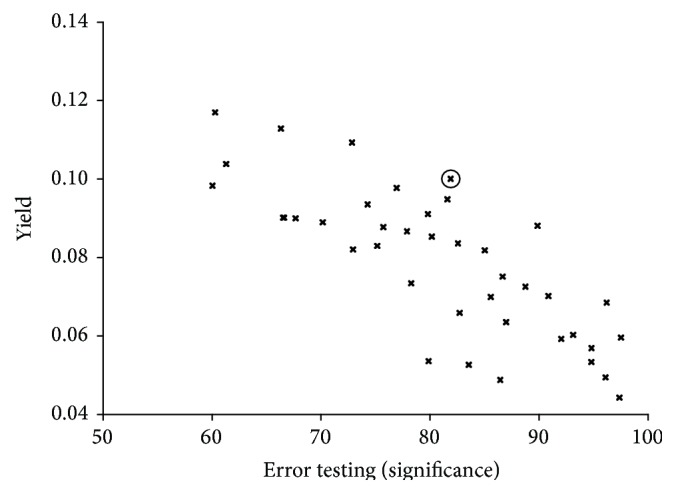
Scatter graph of datasets upon stratified parameter optimization. Identification of optimal parameter set as mtm significance test C90; window size 1024; upper limit *U* = 0.0068359; and *l*sc = 0.0555556 (encircled).

Using the above-defined variations of the parameters for the sc optimization we get 5507 different parameter sets. The complex variations in the command variables, that is, *p* values, significance, and yield of all the parameter sets, are summarized in Figure 1 and Table 2. To identify an optimal parameter set we first introduced a restriction to the *p* value, to safeguard a valid medical evidence of the SCP value. In accordance with the biomedical literature we have chosen a *p* value <0.01 for the further optimization process. After that we executed a two-dimensional binning of the data with respect to the significance and the *p* value. We choose delta for the significance intervals of 1 deg and 0.001 for the *p* value intervals. In contrast to the most binning modes we did not take an average of the yield value for each bin but took the highest yield, respectively, the parameter set in this bin leading to the highest yield. Using this approach, we have achieved retaining of the highest level of sensitivity for SCP per bin. This procedure reduces the amount of parameter set drastically to 40 different sets. The interrelation between significance and yield value of this parameter set is depicted in Figure 2.

Accounting for the need of a significance level for the sc error testing higher than 75% an “optimal” parameter set is clearly found at significance of 81.89% and a yield of 0.10.

The resulting parameter set, consisting of wsize = 1024, mtm stattest = 90%, upper limit *U* = 0.0068359, and *l*sc = 0.0555556, is now used for the optimization of the mean Hilbert phase difference mhpd_pos_.

### 3.1. Hilbert Phase Difference Optimization

**Figure 3:**
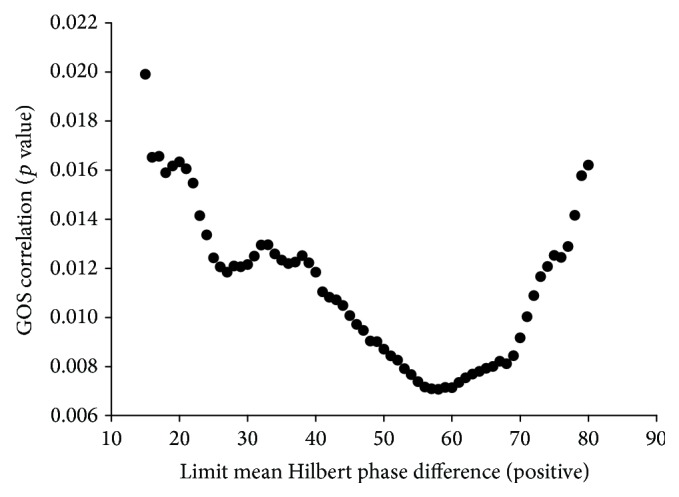
Scatter graph of the correlation between the limit mean Hilbert phase (positive) and patient outcome correlation (*p* value of Pearson correlation SCP with GOS).

**Figure 4:**
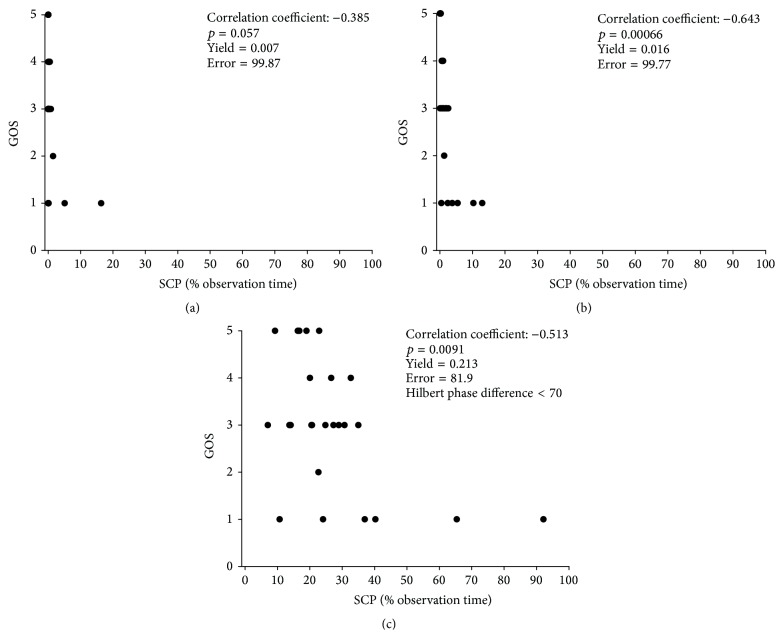
Impact of parameter settings in correlation with patient outcome. (a) Worse scenario with nonsignificant correlation and low yield. (b) Intermediate scenario with improved outcome correlation but low yield resulting in poor data distribution. (c) Optimized scenario with high correlation with patient outcome, maximal yield leading to improved data distribution, and sensitivity for SCP detection.

For the above-mentioned wsize = 1024 we found that *l*mhpd_pos_ is allowed to be a maximum of 70 deg to meet a *α*mhpd_pos_ lower equal to 81.8% and is therefore reproducing an equivalent significance as for the sc calculations. The variations of the *p* value for increasing *l*mhpd_pos_ are depicted in Figure 3. It can be clearly seen that *l*mhpd_pos_ of 70 deg still meets the target of a *p* value <0.01. With this setting for *l*mhpd_pos_ the yield rises to 0.2129. Comparing this result with the yield value reached by the parameter set used in our previous study [18] we find an improvement of yield and therefore an enhancement of the methods sensitivity by a factor of 4.27. To visualize the impact of parameter optimization, we have performed a Pearson correlation between percentage of observation time patients displayed SCP and the clinical outcome, measured by GOS (Figure 4). The three scenarios indicate that depending on the parameter setting the correlation with outcome varies significantly; in addition, sensitivity expressed by yield and accuracy as indicated by error test significance is also highly dependent on the parameter settings.

## 4. Discussion

The prognosis of severe neurological events such as subarachnoid hemorrhage or brain trauma remains exceptionally poor [20, 21]. Despite promising preclinical data, most of the trials prospectively evaluating new therapeutic approaches have invariably failed to demonstrate any significant improvement of outcome [22, 23]. Based on this paucity of causative treatment options, the main intention of modern neurointensive care is to prevent secondary neuronal injury and foster maximal neuronal recovery by adjusting systemic blood pressure, ICP, CPP, and cerebral oxygenation [24, 25]. Since the univariate focus on only one parameter such as ICP has been demonstrated to be unsuccessful [26], it became evident that the entire pathophysiological system following catastrophic neurological injury needs to be monitored and therapeutically adjusted in order to improve patient outcome [27]. Modern approaches in computerized interpretation of multimodal brain monitoring parameter utilizing time series analyses have provided clinical support tools for the real time interpretation of gradual changes in monitoring parameters to unmask systematic changes such as reduced intracranial compliance [18] and disturbed autoregulation [17, 28]. These systems have recently been validated using positron emission tomography [29], microdialysis [30], transcranial Doppler sonography [31], and patient outcome [12, 17], to ensure the predictive value and clinical utility of this approach. As a limitation of our approach, SCP has not been compared to the most established index for disturbed autoregulation (PRx) developed by Czosnyka and coworkers [32]. However, the major task is now to implement these platforms into a prospective clinical trial setting in order to assess the benefit of this approach for treatment adjustment and improvement of patient outcome [7]. Our results clearly demonstrate that SCP detection is highly dependent on the parameter setting. To identify the optimal parameter setting for a future clinical trial employing SCP as real time clinical tool, we have followed three main objectives: (A) high correlation rate to patient outcome in order to avoid false positive SCP detection rates which may lead to overtreatment of patients with potential side effects, (B) maximal sensitivity for SCP occurrence indicated by a high yield (percentage of SCP per observation period), and (C) highest possible accuracy by avoiding autocorrelation events expressed by the significance of the built-in error testing. In conclusion, the proposed parameter optimization stratified to meet the major clinical needs results in a highly specific, sensitive, and reliable method for the detection of intracranial dynamics of patients treated in neurointensive care. We are in the process to utilize these optimized parameters derived from the existing set for a prospective study addressing the clinical usefulness of this approach.

## Figures and Tables

**Table 1:** Parameter ranges utilized for the optimization of SCP analysis tools.

Parameter	Range
Window size	1024, 2048
mtm built-in statistical test significance	50, 90, 95, 99%
Upper limit of frequency interval *U*	0.002–0.008 Hz
*l*sc	All possible values in *U* with *αl*sc <0.4
*l*mhpd_pos_	0–70 degrees

**Table 2:** Range of target variables influenced by parameter optimization.

Parameter	Range
*p* value (outcome correlation)	0.0570–0.0007
Yield	0.1170–0.0047
sig.	60.0445–99.8998
